# A Person-Environment Fit Model to Explain Information and Communication Technologies-Enabled After-Hours Work-Related Interruptions in China

**DOI:** 10.3390/ijerph20043456

**Published:** 2023-02-16

**Authors:** Shanshan Zhang, Fengchun Huang, Yuting Zhang, Qiwen Li

**Affiliations:** School of Management, Zhejiang University of Technology, Hangzhou 310023, China

**Keywords:** person-environment fit, ICT-enabled, after-hours work-related interruptions in China, polychronicity, innovative job performance, in-role job performance

## Abstract

Given the ubiquitous nature of mobile devices and information and communication technologies (ICT), after-hours work-related interruptions (AHWI) occur anywhere and anytime in China. In the current study, an alternative person–environment (P-E) fit model of ICT-enabled AHWI, hereafter referred to as IAWI, that treats polychronic variables as moderated solutions are presented. A cross-sectional survey among 277 Chinese employees (average age: 32.04 years) was conducted in September 2022 and tested by PLS-structural equation modeling to validate our hypotheses. The results indicated that IAWI had a positive influence on employees’ innovative job performance and in-role job performance (β = 0.139, *p* < 0.05; β = 0.200, *p* < 0.01; β = 0.298, *p* < 0.001). Moreover, among employees with higher levels of polychronicity, the heightened effects of IAWI on innovative job performance were increased (β = 0.112, *p* < 0.05). This study offers implications for employees: under IAWI situations, they could search for a person–environment (P-E) that is fit to buffer the negative aspects of IAWI, consequently increasing their innovative job performance and in-role job performance. Future research could extend beyond this framework to explore employees’ IAWI and job performance balance.

## 1. Introduction

Information and communication technologies (ICT) have become a widespread media enabling people to interact with each other anywhere and at any time. ICT can break time and space constraints, specifically during a pandemic such as the COVID-19 outbreak. During a pandemic, people constantly switch back and forth between personal and work roles at home and at work. According to the Work Trend Index released by Microsoft in March 2021, more than 70% of employees want their respective companies to continue providing the option of working remotely from home [1]. Moreover, according to CNNIC 2022 [2], ICT-enabled work is increasingly rising, and ICT users in China have reached 1.027 billion. As a new driving force for economic growth, ICT-enabled telecommuting provides more future possibilities for employees, allowing companies to better adapt to the uncertainties and challenges brought about by sudden crises and digital transformations [3,4]. Nevertheless, flexible working hours also represent the beginning of an unlimited extension of working hours and the disruption of life. That is, given the pervasiveness of ICT, the boundary between work and nonwork/life becomes blurry. Technology-enabled channels have consequences, such as after-hours work-related interruptions (AHWI), which have received much attention from scholars [5].

ICT-enabled AHWI (hereafter referred to as IAWI) refers to work-related occurrences caused by technology during employees’ after-work hours. It breaks individuals’ ongoing nonwork activities and has increasingly become a prevalent work–life interaction form [5]. In general, prior research has used stress–strain-outcome frameworks and indicated the negative consequences of IAWI [6,7,8] (as shown in Table A1). They suggest that ICT invades employees’ personal life and drains personal resources for job demands. For instance, Cheng et al. (2020), who adopted the conservation of resources theory, found that technology-mediated work interruption leads to information overload [9]. Van et al. (2020) and Lanaj et al. (2014) postulated that IAWI results in emotional exhaustion and triggers lower work engagement and less proactive work behavior [10,11].

Nevertheless, given the growing reliance on ICT and the ubiquitous nature of IAWI, people routinely negotiate the boundaries between work and nonwork activities on a daily basis. Work and nonwork domains have become more integrated [12,13,14,15], especially during the COVID-19 period [3,4]. Notably, in China and some Asian countries (e.g., Japan and Korea), the cultural norm of collectivism promotes working after hours [16,17,18]. The Chinese give priority to work in order to benefit their family [19]. From the perspective of individual adaptability, individuals in China tend to adaptively utilize ICT as a means of promoting the integration of work and life. Despite this evidence, previous studies on Chinese employees have generally separately considered work and life, pinpointing that IAWI induces work–life conflict [20]. In particular, less research has explored the IAWI phenomenon from the individual adaptability perspective and examined how IAWI influences Chinese employees’ other behavioral outcomes, e.g., innovative job performance and in-role job performance. Further studies are needed to examine the work and nonwork domains enabled by ICT in China and the relationship between IAWI and its consequences, i.e., other aspects of employees’ behavioral outcomes.

As a key pillar in the organizational behavior field, job performance is defined as employees’ contribution to organizational goals [21]. In the current study, we explore the two types of employees’ behavioral outcomes, i.e., innovative job performance and in-role job performance, for the following reasons. First, in-role job performance and innovative jobs are the main goals sought by organizations, and innovative job performance is specifically not attached to supervisor ratings of performance. Janssen [22] assessed in-role performance in three subdimensions (job description, employee responsibilities, and performance requirements) and evaluated innovative performance by applying three subdimensions (idea generation, idea promotion, and idea realization). Meanwhile, Hagedoorn and Cloodt [23] used three measurements of innovative performance: research and development, patents, and announcements of new products. Second, only a few pieces of research have examined the relationships between IAWI and in-role job performance and innovative job performance, whereas the relationships between IAWI and traditional behavioral outcomes, such as task performance and nonwork performance, have been well established.

According to person–environment (P-E) fit theory, people have innate needs to seek a fit between the external environment and personal demands. In this sense, under IAWI situations, employees may search for a P-E fit that can meet their psychological or practical demands and is adaptable to the imposed ICT-enabled requirements [5,24]. This shift alters employees’ perceptions of IAWI. For instance, IAWI may be deemed positive, necessary, and constructive, providing a flexible and cross-domain environment for employees, consequently increasing their behavioral outcomes [5,25,26]. The P-E fit theory has been used in several digital technology studies and proven positive organizational outcomes, such as organizational commitment and improved task performance [5,24]. In this study, we believe that IAWI may positively influence job performance in line with the P-E fit logic.

Further, individuals routinely negotiate IAWI to accomplish daily work activities. This process of negotiation can be characterized by individual differences. Some employees may be better than others at processing information or dealing with requirements [27]. For instance, prior research has related individuals’ cultural preference differences, such as polychronicity, to ICT-related overload [24]. If an individual prefers to conduct multiple tasks in parallel, then he or she will perceive less information overload. However, how polychronicity matches/fits with IAWI and consequently influences in-role job performance and innovative job performance has not been explored. Above all, the present study is an attempt to bridge the abovementioned research gaps and focus on conceptualization and testing the positive effects of IAWI in China. We adopted a P-E fit lens to explore how polychronicity influences the relationships between IAWI and in-role job performance and innovative job performance. Such an examination may help explore the task-technology fit mechanisms among Chinese employees and cast a new light on the understanding of IAWI, polychronicity, and organizational behavioral outcomes.

The current study aims to (1) investigate IAWI from the P-E fit perspective and (2) incorporate the moderating effect of polychronicity to analyze how it affects the influence of IAWI on employees’ innovative job performance and in-role job performance. We address the deficiency of extant research regarding IAWI by employing a new theoretical perspective, namely, the P-E fit perspective. The time/temporal factor polychronicity is adequately incorporated into the IAWI model to better understand the P-E fit process. This approach is helpful in designing ICT applications that are better fitted to users’ temporal behaviors.

The outline of this study is briefly described as follows. Section 2 introduces the concepts of the P-E fit theory and polychronicity and presents the relationships between IAWI, innovative job performance, in-role job performance, and polychronicity. Section 3 and Section 4 describe the data collection process and concludes the findings. The implications of the findings and future research directions are also illustrated.

## 2. Theoretical Background

### 2.1. Person-Environment Fit Theory

According to P-E fit theory, the person and the environment not only directly affect individual outcomes but also interact with one another to affect these outcomes [28]. Individuals tend to seek out and create “fitting” environments that allow them to manifest their personalities [29]. Past research has established the existence of several types of fits, including the fit between the demands of the environment and personal abilities [30,31], the fit between individual needs and environmental supply [32], and the fit between organizational and personal values [33,34]. Previous studies have also proven a variety of positive outcomes that are linked to P-E fit, such as job satisfaction, organizational commitment, psychological well-being, turnover, job performance, and citizenship behaviors [35,36,37]. Under IAWI situations, employees may search for a P-E fit that can meet their psychological or practical demands and is adaptable to imposed ICT-enabled requirements [5,24]. In this study, we use the P-E fit perspective to explain how individuals can view technology-mediated work interruptions similar to a “fitting” environment, allowing them to experience greater job performance (i.e., in-role job performance and innovative job performance).

### 2.2. Polychronicity

Hall, an anthropologist, proposed the concept of cultural values and discovered polychronic cultures among major Native Americans [38]. In his work, polychronicity refers to an individual’s cultural preference for engaging in two or more tasks or activities simultaneously. Individuals who prefer to engage in multiple tasks simultaneously are polychronic individuals [39]. By contrast, individuals who prefer monochronic time are people who, for example, engage in one task at a time. Furthermore, individuals shape their own individual culture [40], and culture determines their thought processes [41]. Prior experiences are stored in an individual’s mental representations. If the new input matches with memories of past experiences, then it reflects a positive emotion and is said to be congruent [42]. If a mismatch exists between input and stored memory (e.g., individual’s cultural preference), then it is said to be incongruent, and the event does not proceed efficiently.

### 2.3. Hypothesis Development

A number of studies concerning the relationship between IAWI and job performance are limited to examinations of negative influences. However, P-E fit theory suggests that people have innate needs to seek a fit between a person and the environment. Under IAWI circumstances, employees feel urgent and hustled to engage in job tasks. They engage in jobs that are prescribed by specific roles. In contrast to extra-role behaviors that are outside one’s job description and would not be rewarded formally, in-role behaviors are monitored and rewarded [43,44]. Moreover, employees have an achievement-striving motivation to seek and complete in-role tasks even though they are exhausted [45,46]. Specifically, under the higher collectivist culture in China, the availability of IAWI makes employees spend more time, even “24-h”, at work [47]. Following Hofstede (1980), collectivist cultures focus more on achieving group goals [48]. Hu et al. (2022) posit that employees in China, one of the collectivistic countries, consider the threat of IAWI to be low and are dedicated to fulfilling individual tasks [49]. As such, IAWI is expected to lead to higher in-role job performance.

Otherwise, ICT can also be constructive and flexible. For instance, people can use ICT for a variety of purposes, such as making calls, chatting with others, playing games, and surfing the Internet. In the context of IAWI, Chinese employees believe after working hours, that work-related technology use is natural [49], and most of them receive work instructions during nonworking hours [50]. They also tend to creatively apply ICT applications under IAWI situations, allowing them to become more capable of completing tasks. In effect, individuals have an intrinsic motivation to learn and subsequently be rewarded [51]. Resource investments can serve as coping mechanisms to gain potential returns [52]. When employees experience IAWI, they may discover new approaches to innovatively use information technology as a means of increasing productivity [26]. In other words, employees invest resources to cope with IAWI, view IAWI from the positive side [52,53,54], and consequently achieve innovative job performance.

On the basis of the discussion above, we hypothesize the following:

**H1:**  *IAWI is positively associated with in-role job performance in China.*

**H2:**  *IAWI is positively associated with innovative job performance in China.*

Prior research has related polychronicity to ICT-related overload [24]. If an individual prefers to conduct multiple tasks in parallel, then he or she perceives less information overload. In this sense, if employees’ polychronicity scores increase, then they may be more adaptive to IAWI circumstances, which may facilitate the work to be accomplished. König and Waller [55] and Bluedorn [56] also argue that polychronicity shares a common meaning with multitasking. Although the explosion of ICT has increased the incidence of multitasking [57], polychronics believe that ICT also helps them to efficiently handle multiple tasks [57]. Hence, a match exists between IAWI and the individual’s cultural preference, that is, polychronicity. For Chinese employees needing to deal with ICT-enabled demands, if they are polychronic, then the event or task will proceed efficiently [27], resulting in increased job performance. We expect polychronicity to strengthen the relationship between Chinese employees’ perceptions of IAWI and their job performance (i.e., in-role job performance and innovative job performance).

Thus, we hypothesize the following:

**H3:**  *Polychronicity positively moderates the positive effect of IAWI on in-role innovative job performance in China.*

**H4:**  *Polychronicity positively moderates the positive effect of IAWI on innovative performance in China.*

**Figure 1:**
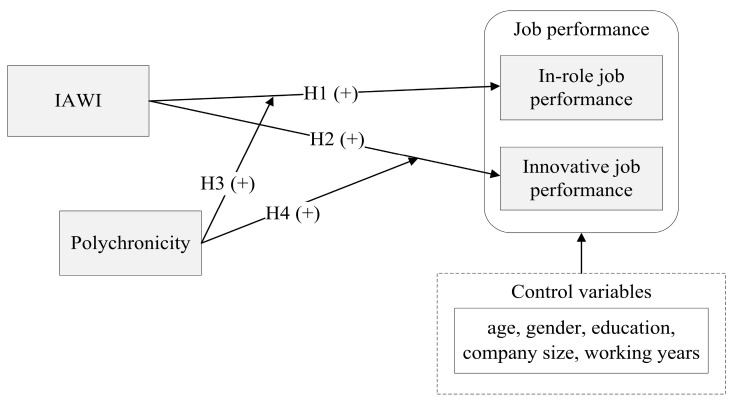
Proposed research model.

In summary, our study focuses on exploring the positive impacts of IAWI on in-role job performance and innovative job performance among individuals with different polychronicity values. Polychronicity is adequate for use as a moderator in the IAWI model. Several control variables, such as age, gender, educational background, income, industry, company size, working years, and occupation ranking, are included to account for in-role job performance and innovative job performance [5,58]. The proposed model is shown in Figure 1.

## 3. Methodology

A cross-sectional survey design and employees across China were both considered in the empirical testing of the proposed research model. Before sending out the survey, the was piloted among ten employees and reviewed by three researchers. The questionnaire was distributed to them to check if the measurements of IAWI, polychronicity, in-role job performance, innovative job performance, and control variables were readable. Some modifications were made according to their feedback. Then, the questionnaire links were distributed to participants via a professional data collection agency in China: Sojump. The links lasted for one week, and the participants were offered a monetary incentive of 15 RMB for completing the survey. The agency offers a service that filters out non-working people. In particular, 311 people participated in the survey. There was one question about the subjects’ current status (What is your current status? 1. Students; 2. Part-time work; 3. Full-time work; 4. Unemployed). A total of 34 people who were not employees were excluded. Finally, a total of 277 employees were used for the subsequent data analysis.

Measurements of the independent and dependent variables were adapted from the literature (see Table A2). In particular, the measurement items for IAWI were adapted from Chen and Karahanna [5]. For instance, employees who have IAWI are those who are interrupted about work-related matters through technology, such as Wechat, Dingding, and so on, during their after-work time. The measurements for in-role job performance and innovative job performance were based on Williams and Anderson [59] and Janssen and Van Yperen [60], respectively. If employees completed the duties specified in their job descriptions and often searched out new working methods, these people had higher levels of in-role job performance and innovative job performance. Polychronicity was measured using the scales developed by Slocombe and Bluedorn [61]. Polychronic people prefer multitasking. All items, as detailed in Table A2, were measured on seven-point Likert-type scales, ranging from “strongly disagree” to “strongly agree.”

As our study was conducted among Chinese employees, we used the back-translation method to translate the original English survey instruments into Chinese [62]. In particular, four bilingual researchers initially translated the questionnaire into Chinese, and then another bilingual researcher translated the version back into English. After obtaining their feedback, some changes were incorporated, and then the links were sent out for pretest and formal data collection.

## 4. Analyses and Results

### 4.1. Statistical Data Analysis

Descriptive statistics are presented to show how the participants were distributed across all the control categories. Factors analysis is used to validate the reliability and validity of the constructs for the present study. In the correlation analyses, we examined the relationships between IAWI, polychronicity, in-role job performance, and innovative job performance.

The structural equation modeling method has been well established for assessing measurement models and structural models, and it is advantageous for theory development [63,64,65,66]. In view of the exploratory nature of the study, PLS-structural equation modeling was used to test the research model in SmartPLS 2.0 M3 (SmartPLS GmbH, Oststeinbek, Germany) [67]. Specifically, constructs’ reliability and validity analyses were conducted by performing the PLS algorithm program. The estimation of the path coefficients was computed by performing a standard mediation bootstrap resampling program via SmartPLS 2.0 M3 (SmartPLS GmbH, Oststeinbek, Germany).

### 4.2. Profile of Respondents

Table 1 shows the respondents’ demographics. Among the 277 participants, 141 (50.9%) were males, and 136 respondents (49.1%) were females, with an average age of 32.04 years (SD = 14.82). Most of them had a bachelor’s educational background (mean = 5.36; 1 = less than high school/secondary school; 2 = high school/secondary school; 3 = associate degree/higher diploma; 4 = bachelor’s degree; 5 = master’s degree; 6 = doctorate/PhD). On average, 225 (81.2%) respondents earned between 5001 and 20,000 RMB (mean = 3.46; SD = 0.88). The respondents mainly worked in the fields of internet/information systems, construction/manufacturing, and retail trade (mean = 4.67; SD = 3.09); those people are assumed to be reasonably representative of ICT users in China [2]. The vast majority of the respondents (49.1%) were employees (mean = 1.81; SD = 0.92), and 50.9% were basic-level managers, middle-level managers, senior leaders, and others (mean = 4.67; SD = 3.09). At least half of the respondents (47.7%) had worked for their company for more than 5 years and no more than 10 years (mean = 3.75; SD = 0.96). At least half of the companies (49.5%) employed 100–500 individuals.

### 4.3. Results of Assessing the Measurement Model

While common method variance is considered a concern in behavioral research [68], we addressed the potential issue of common method bias by performing three tests. First, the Harmon single-factor test was executed [69]. The results revealed that four factors were selected among the 16 components, and the first factor could explain only 26.48% of the total variance (as shown in Table 2). This number is below the threshold (i.e., 50%), which indicates that the study does not have a substantial common method bias problem. Second, following the work of Liang and Saraf [70], a common method factor analysis was included to estimate variances of all the principal constructs’ indicators. The average method-based variance was 0.003 (Table 3), which is much smaller than the average substantively explained variance of indicators (i.e., 0.621). Moreover, the substantive factor loadings of each indicator are statistically significant and larger than the method factor loadings. Third, according to Lowry and Gaskin [64] and Pavlou, Liang [71], if any correlation of constructs reached 0.9, then the survey had a common method bias problem. None of the correlations exceeded 0.9 (see Table 4); hence, our data did not suffer from common method bias. Thus, a common method bias is not a concern for this study.

Then, we tested the internal consistency of the sales. The values of composite reliability and Cronbach’s α all exceeded 0.7 (see Table 4), demonstrating high levels of reliability on the scales [72]. In addition, the convergent and discriminant validity of the constructs were examined via confirmatory factor analysis [73,74]. As shown in Table 4, the square root of the average variance extracted (AVE) was greater than its correlation with other variables, all AVE values exceeded 0.5, and all loadings exceeded 0.6 (see Table 3), indicating the acceptable discriminant validity and convergent validity [75,76,77] of the present study. The general mean and standard deviation characteristics of the constructs are also presented in Table 4.

### 4.4. Results of the Analysis of the Path Model

We performed a standard bootstrap resampling procedure in SmartPLS 2.0 M3 to test the proposed model [63]. The results indicate the positive significant influence of IAWI on in-role job performance and innovative job performance (β = 0.139, *p* < 0.05; β = 0.200, *p* < 0.01; β = 0.298, *p* < 0.001), supporting H1 and H2. Age, gender, education, industry, company size, working years, and occupation ranking had no influence on in-role job performance and innovative job performance. However, income is significantly positively related to in-role job performance and innovative job performance (β = 0.152, *p* < 0.05; β = 0.170, *p* < 0.05). Polychronicity has a positive moderating effect on the relationship between IAWI and innovative job performance (β = 0.112, *p* < 0.05), but the enjoyment has no moderation effect on the relationship between IAWI and innovative job performance (β = −0.093, *p* > 0.05). Therefore, H4 is supported, whereas H3 is not supported.

**Figure 2:**
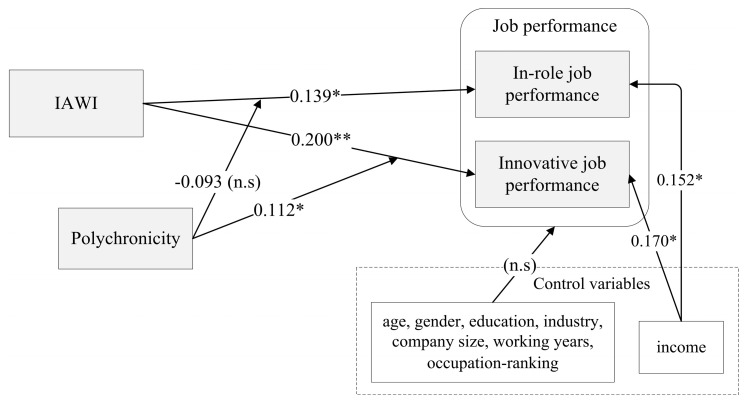
Results of path-model analysis. * *p* < 0.05, ** *p* < 0.01.

In summary, H1, H2, and H4 are significant at the 0.05, 0.01, and 0.001 levels, respectively (Figure 2). Table 5 presents the path coefficients (β) and significance (*t* values) of the research model.

## 5. Discussion of the Findings

**Figure 3:**
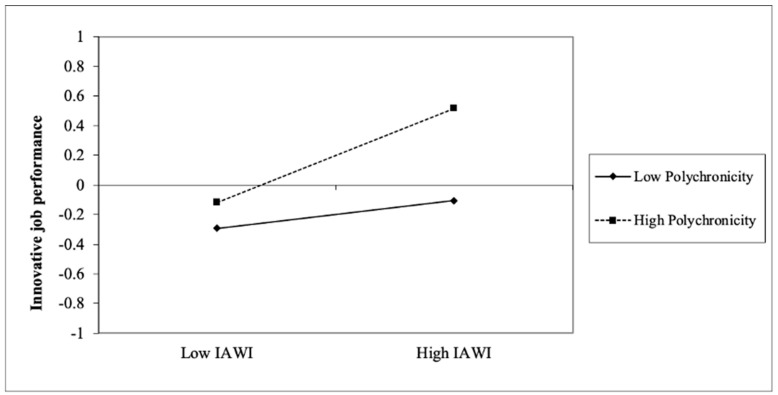
The moderating effect of polychronicity on the relationship between IAWI and innovative job performance.

The aim of this study was to examine how IAWI and polychronicity influence employees’ in-role job performance and innovative performance. The results confirm the proposed positive effects of IAWI on in-role job performance and innovative job performance and the positive moderating effect of polychronicity on the relationship between IAWI and innovative job performance. As shown in Figure 3, the relationship between IAWI and innovative job performance strengthens as polychronicity increases. In particular, when polychronicity is high, IAWI and polychronicity can be complementary when performing innovative job activities. When polychronicity is low, users have low intentions or habits to perform multitasking activities, and the path coefficient from IAWI to innovative job performance is weakened. Thus, H4 is supported.

Notably, polychronicity has no interaction effect on the relationship between IAWI and in-role job performance. This phenomenon may be attributed to employees adapting to IAWI situations in most cases. This P-E fit enables individuals to better deal with their moment-to-moment in-role tasks, which nurtures routines among Chinese employees. Polychronicity is less likely to affect the correlation from IAWI to in-role job performance. By contrast, polychronic individuals have greater latitude to leverage available resources; thus, people may choose to deal with IAWI situations through multitasking, which may lead to higher innovative activity handling. Future research may analyze the IAWI and in-role job performance phenomenon under varying personal circumstances. Another interesting finding is that income is positively correlated with in-role job performance and innovative job performance. This finding may be explained by income, which is a major concern for most people. Financial stress largely pushes individuals to work harder and be innovative when performing job tasks.

In summary, the findings prove the viability of the proposed hypotheses except for H3. This study may provide empirical support for examining the consequences of IAWI.

## 6. Theoretical Contribution and Practical Implications

First, this study helps to examine the relationships between IAWI and its consequences, i.e., in-role job performance and innovative job performance. In essence, interruption management is a common challenge faced by present-day employees. However, previous studies have demarcated work and life as two distinct spheres [13,78], focusing largely on employees’ work performance and nonwork performance. This study enriches the existing literature by revealing that IAWI has positive influences on other aspects of Chinese employees’ behavioral outcomes, i.e., in-role job performance and innovative job performance. We identified that IAWI could be deemed necessary and constructive, providing a flexible and cross-domain environment for Chinese employees, consequently increasing their behavioral outcomes.

Second, this study develops an alternative model for investigating IAWI through the P-E fit lens. Previous studies have generally addressed the negative outcomes of IAWI [79,80]. In particular, they focused on exploring the negative connotation of interruptions, i.e., increased emotional exhaustion and decreased task performance correlating with interruptions from a negative perspective [10,11]. However, interruptions may not always be counterproductive, as they may even yield desirable gains among Chinese employees from a positive perspective [26]. IAWI can be viewed as a mere activity or behavior that helps employees achieve organizational tasks and self-fulfillment [5,53,54,81]. The results of our study validate that, under IAWI situations, Chinese employees may search for a P-E fit that could meet their psychological or practical demands and is adaptable to the imposed ICT-enabled requirements. This may provide new insights for understanding employees’ perceptions of IAWI and provide new directions for future IAWI research.

Third, the results show that polychronicity and IAWI can work together to improve innovative job performance. Previous research has recognized the importance of polychronicity in dealing with information overload in the ICT context [24]. Polychronicity also plays an important moderating role in enhancing employees’ innovative job performance. Therefore, individuals and organizations must understand the role of individual differences as a means of effectively leveraging differences, thus promoting the positive effects of IAWI on innovative job performance. In summary, our study contributes an overall comprehensive understanding of IAWI and innovative job performance by combining P-E and individual difference/preference perspectives.

Finally, the results of this study have implications for practice. In particular, drawing on P-E theory, organizations and enterprises should provide more supporting regulations/policies, ICT facilities, and equipment to improve the matching and balance of people and the environment. Websites can provide a favorable and innovative ICT interface for users to help them cope with after-hours work-related tasks. For instance, preset text messages (e.g., “I will deal with it”), as an autonomous tool, can be set up for users to manage interruptions. In using this tool, technological solutions allow individuals with IAWI experiences to manage interruptions and be productive in their work.

## 7. Limitations and Future Research Directions

We acknowledge that this study has limitations. First, this study used a cross-sectional survey and self-reported data. As a result, people may overestimate their job performance. Future research can address this issue by conducting longitudinal field studies to track individuals’ actual behaviors over time. Second, the effects of IAWI may differ across countries; future cross-cultural studies can help assess the effects of cultural differences on IAWI. Third, we focused on the direct effect of IAWI on in-role job performance and innovative job performance. Future research may investigate other relevant mediators and moderators to further contextualize people’s P-E fit process under IAWI situations. Finally, the sample size of our study is relatively small, and a larger sample in larger areas could be selected for modeling tests in the future.

## 8. Conclusions

Cross-domain interruptions are unavoidable because of the increasing ubiquity of present-day mobile technologies. Prior studies have focused on the detrimental effects of IAWI. In this study, we highlighted the difference by concentrating on individuals’ P-E fit capabilities and needs. As hypothesized, Chinese employees tended to venture beyond the negative aspects of IAWI and were more inclined to complete in-role tasks and innovative job performance. Polychronicity and IAWI complement one another in improving innovative job performance. In conclusion, our study is insightful for future ICT-related research and has implications for individuals, organizations, and ICT developers.

## Figures and Tables

**Table 1:** Demographic characteristics of respondents (n = 277).

Variables	Frequency (Percent)		Mean (Standard Deviation)
Age (enter age):			32.04 (14.82)
Gender:			1.49 (0.50)
Male	141	50.9%	
Female	136	49.1%	
Education:			4.05 (0.69)
Less than high school/secondary school	2	0.7%	
High school/secondary school	5	1.8%	
Associate degree/Higher Diploma	23	8.3%	
Bachelor’s degree	204	73.6%	
Master’s degree	34	12.3%	
Doctorate/Ph.D.	9	3.3%	
Income			3.46 (0.88)
Below 3500	4	1.4%	
3501–5000	22	7.9%	
5001–10,000	125	45.1%	
10,001–20,000	100	36.1%	
20,001–50,000	20	7.2%	
Above 50,001	6	2.2%	
Industry			4.67 (3.09)
Agriculture, forestry and fishing	6	2.2%	
Internet/information systems	93	33.6%	
Construction/manufacturing	49	17.7%	
Transportation, storage, postal and courier services	20	7.2%	
Import/export and wholesale	15	5.4%	
Retail trades	11	4%	
Financial, insurance and real estate activities	29	10.5%	
Accommodation and food service activities	14	5.1%	
Public administration	11	4%	
Education	12	4.3%	
Health	4	1.4%	
Others	13	4.7%	
Company size:			2.29 (0.92)
Below 100	50	18.1%	
100–500	137	49.5%	
501–1000	51	18.4%	
Over 1000	139	14.1%	
Working years			3.75 (0.96)
Below 1 year	5	1.8%	
1–3 years	29	10.5%	
3–5 years (not include 3 years)	53	19.1%	
5–10 years (not include 5 years)	132	47.7%	
Above 10 years	58	20.9%	
Occupation-ranking			1.81 (0.92)
Employee	136	49.1%	
Basic-level manager	66	23.8%	
Middle-level manager	68	24.5%	
Senior leadership	5	1.8%	
Other	2	0.7%	

**Table 2:** Total variance explained.

Component	Initial Eigenvalues		
	Total	Variance (%)	Cumulative (%)
1	4.237	26.482	26.482
2	2.292	14.326	40.808
3	1.835	11.47	52.277
4	1.752	10.953	63.23
5	0.894	5.586	68.816
6	0.832	5.199	74.015
7	0.668	4.175	78.19
8	0.573	3.583	81.773
9	0.545	3.408	85.181
10	0.475	2.969	88.151
11	0.426	2.66	90.811
12	0.395	2.467	93.278
13	0.374	2.34	95.618
14	0.296	1.851	97.47
15	0.217	1.355	98.825
16	0.188	1.175	100
	**Extracted sums of squared loadings**		
1	4.237	26.482	26.482

**Table 3:** Common method bias analysis and factor loadings of measurement items.

Construct	Item	Factor Loading	Substantive Factor Loading (R1)	R1^2^	Method Factor Loading (R2)	R2^2^
Information and communication technologies enabled after-hours work-related interruptions (IAWI)	IAWI1	0.884	0.7678	0.590	0.0290	0.001
	IAWI2	0.859	0.7018	0.493	0.0657	0.004
	IAWI3	0.617	0.8287	0.687	−0.0582	0.003
	IAWI4	0.602	0.7916	0.627	−0.0295	0.001
In-role job performance (RJP)	RJP1	0.769	0.7221	0.521	−0.0121	0.000
	RJP2	0.677	0.7403	0.548	−0.0284	0.001
	RJP3	0.660	0.6515	0.424	−0.0026	0.000
	RJP4	0.697	0.8247	0.680	−0.0954	0.009
	RJP5	0.769	0.6873	0.472	−0.0002	0.000
	RJP6	0.708	0.6882	0.474	0.1274	0.016
Innovative job performance (IJP)	IJP1	0.837	0.8662	0.750	−0.0470	0.002
	IJP2	0.800	0.8429	0.710	−0.0818	0.007
	IJP3	0.819	0.7765	0.603	0.0680	0.005
	IJP4	0.787	0.7606	0.579	0.0575	0.003
Polychronicity (POL)	POL1	0.909	0.9466	0.896	0.0247	0.001
	POL2	0.969	0.9388	0.881	−0.0247	0.001
Average				0.621		0.003

**Table 4:** Means, standard deviations, Cronbach’s α, CR, AVE, and construct correlations.

	Mean	Standard Deviation	Cronbach’s α	CR	AVE	1	2	3	4
1. IAWI	5.07	1.14	0.776	0.840	0.566	**0.752**			
2. RJP	5.92	0.70	0.812	0.859	0.511	0.125	**0.715**		
3. IJP	5.59	0.89	0.823	0.883	0.657	0.190	0.400	**0.811**	
4. POL	4.37	1.57	0.875	0.937	0.883	−0.020	0.125	−0.075	**0.940**

CR = composite reliability, IAWI = information and communication technologies enabled after-hours work-related interruptions, RJP = in-role job performance, IJP = innovative job performance, POL = polychronicity. The diagonal elements (in bold and underlined) are the square roots of the average variance extracted (AVE) values.

**Table 5:** Summary of path coefficients and significant levels.

Tested Path	Path Coefficient (β)	*t*-Value (df = 277)	Hypothesis Supported?
Hypotheses			
H1. IAWI → In-role job performance	0.139	2.020 *	Yes
H2. IAWI → Innovative job performance	0.200	3.013 **	Yes
H3. IAWI * Polychronicity → In-role job performance	−0.093	1.762	Not supported
H4. IAWI * Polychronicity → Innovative job performance	0.112	2.143 *	Yes
**Covariates**			
Age → In-role job performance	0.041	0.874	
Gender → In-role job performance	0.089	0.523	
Education → In-role job performance	−0.018	0.401	
Income → In-role job performance	0.152 *	1.971	
Industry → In-role job performance	−0.052	1.062	
Company size → In-role job performance	−0.055	1.101	
Working years → In-role job performance	0.127	1.545	
Occupation-ranking → In-role job performance	−0.089	1.421	
Age → Innovative job performance	−0.031	0.303	
Gender → Innovative job performance	0.035	0.842	
Education → Innovative job performance	−0.092	1.509	
Income → Innovative job performance	0.170 *	2.284	
Industry → Innovative job performance	−0.056	1.139	
Company size → Innovative job performance	0.068	1.322	
Working years → Innovative job performance	−0.009	0.135	
Occupation-ranking → Innovative job performance	0.123	1.802	

* *p* < 0.05, ** *p* < 0.01.

## Data Availability

The data of this study will be available from the first and corresponding authors upon reasonable request.

## Appendix A

**Table A1:** Summary of Technology-Mediated Work Interruptions Related Research.

Study	Method	Context	Theories Used	Constructs in the Model
[24]	Survey	Use of mobile phones	N/A	Polychronicity, age, memories of past cognitive overload, memories of past emotional overload, information and communication technology-related overload
[82]	Survey	Work-home segmentation or integration	P-E fit theory	Work-family conflict, work-home conflict stress, segmentation preferences, supplies, job satisfaction
[58]	Survey	Social media usage	Uses and gratification theory, affordances theory	Social use, hedonic use, cognitive use, social capital (number of expressive ties, number of instrumental ties, relational dimension, cognitive dimension), job performance
[5]	Survey	Life interrupted	N/A	Interruption overload, after-hours work interruptions, work performance, work exhaustion
[6]	Survey	Use of a mobile device for work during family time (mWork)	Conservation of resources (COR) theory and family systems theory	mWork, job incumbent time-based work–life conflict (WFC), job incumbent strain-based WFC, job incumbent behavior-based WFC, incumbent, spousal resentment towards job incumbent’s organization, job incumbent organizational commitment, spousal commitment to job incumbent’s organization, job incumbent turnover intentions
[7]	Quantitative and qualitative research methodologies	E-mail as a stress in people’s life	N/A	Total hours worked, overload, coping, number of e-mails
[8]	Survey	Use of communication technologies beyond normal work hours	Boundary theory and related research on role integration	Communication technologies use after hours, affective commitment, job involvement, ambition, employee work-to-life conflict
[9]	Survey	Information technology-mediated interruptions	Conservation of resources theory	Congruent IT-mediated information interruption, incongruent IT-mediated information interruption, sequential processing, sequential processing, interruption overload, emotional exhaustion
[83]	Survey	Technology-mediated cross-domain interruptions	N/A	Technology mediated work-to-nonwork interruptions, work-to-nonwork conflict, nonwork performance, technology mediated nonwork-to-work Interruptions, nonwork-to-work conflict, work performance
[84]	Survey	Mobile usage	Rational actor theory	Level of uncertainty, cost/benefit evaluation, predicted interruption value, interruption response decision
[85]	Survey	Work–life Balance	Conservation of resources theory	Supervisors’ technology-mediated interruption behavior, information overload, sense of control, work/non-work exhaustion, work/non-work performance, supervisors’ work–life balance
[86]	Qualitative research methodology	Technology mediated work–life	Utilization of sociomaterial theory	Work–life balance, boundary management, technology, sociomateriality, power distance, collectivism
[87]	Survey	Conflict and quality of life in the digital age	Conservation of resources theory	Frequency of interruptions outside of work, frequency of interruptions at work mediated by technology, conflicts outside of work, conflicts at work, performance at work, performance outside of work
[88]	Survey	Online interruptions on task performance	Information richness theory	Perceived interruption, task performance, the rate’s types of interruptions, richness of interruption, the interruption rate on task performance
[89]	Survey	Telework	N/A	Fixed site telework, mobile telework, flexiwork, individual characteristics, organizational and technological contexts, the impacts on their work
[90]	Survey	Mobile devices in older users	Inhibitory deficit theory	Age, demands from technology-mediated interruptions, role-based stress, use of mobile technology for work

**Table A2:** Measurement Scales (All scales were measured as 7-point Likert-type scales ranging from strongly disagree to strongly agree).

Construct	Source	Items
Information and communication technologies enabled after-hours work-related interruptions	Adapted from Chen and Karahanna [5]	To what extent do you agree or disagree with the following? AHWI1: During my cyber-life, I frequently get interrupted about work-related matters through technology (by phone, e-mail, messaging, Dingding, WeChat). AHWI2. I frequently stop what I am doing during my cyber-life to initiate work-related activities through technology (by phone, e-mail, messaging, Dingding, WeChat). AHWI3. During my cyber-life, dealing with work-related interruptions initiated by others (by phone, e-mail, messaging, Dingding, WeChat) is time-consuming. AHWI4. Dealing with work interruptions I initiate during my cyber-life (by phone, e-mail, messaging, Dingding, WeChat) is time-consuming.
In-role job performance	Adapted from Williams and Anderson [59]	To what extent do you agree or disagree with the following? RJP1. I adequately complete assigned duties during my in-role job. RJP2. I fulfill responsibilities specified in the job description during my in-role job. IJP3. I always complete the duties specified in my job description. IJP4. I meet all the formal performance requirements of the job. IJP5. I fulfill all responsibilities required by this job. IJP6. I successfully perform essential duties.
Innovative job performance	Adapted from Janssen and Van Yperen [60]	How often do you perform the following work activities? IJP1.I often create new ideas for improvements. IJP2. I often mobilize support for innovative ideas. IJP3. I often search out novel working methods. IJP4. I often transform innovative ideas into useful applications.
Polychronicity	Adapted from Slocombe and Bluedorn [61]	To what extent do you agree or disagree with the following? PLO1.I like to juggle several activities at the same time. PLO2.I like to multi-task.
